# Local Immune Stimulation by Intravesical Instillation of Baculovirus to Enable Bladder Cancer Therapy

**DOI:** 10.1038/srep27455

**Published:** 2016-06-08

**Authors:** Wei Xia Ang, Ying Zhao, Timothy Kwang, Chunxiao Wu, Can Chen, Han Chong Toh, Ratha Mahendran, Kesavan Esuvaranathan, Shu Wang

**Affiliations:** 1 Department of Biological Sciences, National University of Singapore, 117543 Singapore; 2 Institute of Bioengineering and Nanotechnology, 138669 Singapore; 3 Tessa Therapeutics, 239351 Singapore; 4 Division of Medical Oncology, National Cancer Centre, 169610 Singapore; 5 Department of Surgery, Yong Loo Lin School of Medicine, National University of Singapore, 119074 Singapore.

## Abstract

Intravesical instillation of Bacillus Calmette-Guérin is currently used as adjuvant therapy for superficial, non-muscle invasive bladder cancer (NMIBC). However, nearly 40% of patients with NMIBC will fail Bacillus Calmette-Guérin therapy. In an attempt to investigate the feasibility of using insect baculovirus-based vectors for bladder cancer therapy, we observed that intravesical instillation of baculoviruses without transgene up-regulated a set of Th1-type of cytokines and increased the survival rate of mice bearing established orthotopic bladder tumors. When baculoviral vectors were used to co-deliver the mouse CD40 ligand and IL-15 genes through intravesical instillation, the immunogene therapy triggered significantly increased bladder infiltrations of inflammatory monocytes, CD4^+^, CD8^+^ and γδ T lymphocytes. All treated animals survived beyond 12 months whereas control animals died around 2 months after tumor inoculation. We conclude that direct intravesical instillation of baculoviral gene transfer vectors holds the potential to be a novel therapeutic modality for NMIBC.

Adjuvant therapy is often given following transurethral resection of superficial, non-muscle invasive bladder cancer (NMIBC) to control tumor recurrence and progression^1^,^2^. Intravesical immunotherapy with Bacillus Calmette-Guérin (BCG) is currently recommended for patients with intermediate- or high-risk of bladder cancer recurrence. BCG is produced from attenuated live bovine tuberculosis bacterium and can activate innate immune responses mediated by cytokines such as interleukin-2 (IL-2), IL-12, IL-18 and interferon-gamma (INF-γ). Unfortunately, up to 40% of patients with NMIBC will fail BCG immunotherapy within the first year due to BCG refractory, resistance, relapsing, or intolerance^1^,^2^,^3^. Currently, there is still no gold standard for salvage therapy after BCG failure, highlighting the urgent need to develop new adjuvant therapies to improve treatment outcomes for patients with NMIBC.

The bladder is a hollow organ, allowing nonsurgical intravesical drug administration through a urethral catheter and evaluation of treatment efficacy by the means of endoscopy. Taking advantage of the fact that intravesically delivered therapeutics act locally with limited systemic exposure and that superficial bladder cancer is easily accessible, viruses, either replication-competent viruses or replication-deficient recombinant viral vectors, have been tested for bladder cancer therapy in tumor models^3^,^4^. These viral therapeutics include vaccinia virus^5^,^6^,^7^,^8^, adenovirus^7^,^9^,^10^,^11^,^12^,^13^,^14^,^15^, canarypox virus^7^, reovirus^16^, retrovirus^17^,^18^,^19^,^20^, lentivirus^21^, and vesicular stomatitis virus^22^. At least 6 clinical trials have been reported on the use of vaccinia virus and adenoviral vectors to treat bladder cancer^4^,^6^,^11^,^12^,^14^,^15^. However, the hurdles to translating the current success of these viral therapeutics to a broad clinical application appear high, which include the lack of gene delivery vectors with active immunostimulatory function and inefficient expression of a therapeutic gene in bladder tumor cells and in the organ.

Recombinant vectors derived from the insect baculovirus *Autographa californica multiple nucleopolyhedrovirus* (AcMNPV) hold the ability to enter mammalian cells without replicating or causing toxicity to the transduced cell. Since baculoviral vectors with a mammalian expression cassette could be highly effective in mediating transient expression and usually do not integrate into the genome of the transduced cells^23^, they are ideally suited for applications requiring short-term, high level transgene expression and pose much less risk of insertional mutagenesis. From an application point of view, baculoviral vectors are easy to manipulate, able to carry large (at least 38 kbp) and multiple DNA inserts, and can be readily produced in serum-free cell culture medium and purified at high titers in Biosafety Level 1 laboratories. Thus, recombinant baculoviruses have been suggested as a novel type of vectors for cancer gene therapy^23^,^24^,^25^,^26^,^27^.

In preclinical animal studies, baculoviral vectors have been shown to possess a strong adjuvant activity in inducing humoral and cellular immune responses against co-administered antigens, possibly through promoting maturation of dendritic cells (DCs) and producing pro-inflammatory cytokines, chemokines and type I IFNs^28^,^29^ and activating natural killer (NK) cell-dependent antitumor immunity^30^,^31^. The “adjuvant” effect of baculoviral vectors is attributed to the high frequency of CpG motifs in the viral genome, which is similar to that in bacterial DNA and significantly higher than that of mammalian and adenovirus DNA^32^,^33^. However, a critical obstacle toward *in vivo* baculoviral transduction is the inactivation of systemically delivered baculoviral vectors as a consequence of virus recognition by serum complement proteins, a major component of the innate immune system^34^. Hence, in immunocompetent animals systemically delivered baculoviral vectors failed to transduce target cells.

Bladder cancer therapy can be performed with intravesical catheterization through the urethra and easily avoids many barriers to systemic virus administration, thereby presenting a unique opportunity to explore *in vivo* applications of baculoviral vectors. Herein we report that intravesical treatment with baculoviruses alone is able to prolong survival of mice with established orthotopic bladder cancer. We further demonstrate that baculoviral vectors are effective in delivering therapeutic genes into normal and malignant urothelial cells and can be used for immunogene therapy for bladder cancer in a mouse model.

## Materials and Methods

### Baculoviral vectors

Recombinant baculoviral vectors, CMV-Luc, CMV-Luc-WPRE and CMV-RU5-Luc-WPRE, were constructed using BAC-to-BAC baculovirus expression system (Invitrogen, Carlsbad, CA). BV-CD40 ligand (CD40L) virus and BV-IL15 virus which contain the mouse CD40L gene and mouse IL15 gene respectively were produced by homologous recombination after co-transfection of *Sf9* insect cells with pBacPAK6 transfer vector containing the expression cassette and linearized AcMNPV viral DNA (Clontech, Mountain View, CA). BacPAK6, the parental virus with the lacZ gene driven by viral polyhedrin promoter, was obtained from Clontech.

### Mouse tumor model

Adult female C57BL/6 mice were used to generate orthotopic bladder tumors on the luminal surface of the bladder by intravesical instillation of syngeneic MB49 cells with a 24-gauge catheter. After a 30-minute PLL pre-treatment, 100 μl of MB49 cells in PBS was instilled and retained in the bladder for 1 hour by leaving the catheter *in situ* and clamped. A dose of 1 × 10^5^ cancer cells per animal was used in all experiments. Thereafter, the catheter was removed and the bladder was evacuated by spontaneous voiding. MB49 cells used for tumor inoculation were pre-labeled with a lipophilic, near-infrared fluorescent dye DiR (20 ng/ml) (Caliper Life Sciences) overnight. One week after intravesical instillation of MB49 cells, mice were imaged with IVIS100 *in vivo* imaging system using 710 nm excitation and 760 nm emission filter set to examine tumor implantation. Animals successfully implanted with DiR-labeled MB49 cells and with similar tumor burden were kept for experiments designed to evaluate therapeutic efficacy. In the current study, the fluorescent signal of the DiR-labelled MB49 cells persisted without substantial change for at least 7 days. The signal began to decay thereafter, although remaining weakly detectable even on day 14. Hematuria, an indicator for the tumour formation in the bladder, was also monitored in some experiments to further confirm the tumor implantation and growth.

To test therapeutic effects of baculoviral vectors, mice bearing bladder tumors were randomized to control or treatment groups, re-anaesthetized, and re-catheterized. Baculoviruses (1 × 10^8^ pfu in 100 μl), PBS (100 μl), or BCG (1.35 mg = 2.3 × 10^7^ colony forming units in 100 μl, Aventis Pasteur, Canada) were intravesically instilled and retained in the bladders for 2 hours. In some experiments, mice were given up to three weekly intravesical instillations. Animals were observed for up to 12 months for signs and symptoms of bladder cancer (hematuria and weight loss) and viability status.

The animal study protocol was reviewed and approved by Institutional Animal Care and Use Committee (IACUC), the Biological Resource Centre, the Agency for Science, Technology and Research (A^*^STAR), Singapore (Permit Number: BRC IACUC 110612). The methods were carried out according to the guidelines for the Care and Use of Animals for Scientific Purposes issued by the National Advisory Committee for Laboratory Animal Research, Singapore.

### Cytokine/chemokine expression

Mice were euthanized 48 hours after intravesical instillation of PBS or BacPAK6. Bladders were harvested and weighed. Bladder homogenates were obtained by adding 1 ml of a tissue lysis buffer (Fermentas, Maryland, USA) with a protease inhibitor cocktail (Merck, Darmstadt, Germany) to 50 mg of tissue sample and homogenizing by sonication. Bladder homogenates were then centrifuged at 16,000 × g for 30 min at 4 °C and the supernatants collected. Protein concentrations of the supernatants were determined by the Biorad protein assay method (Biorad, California, USA). An aliquot of the supernatant containing 80 μg of total protein concentration was loaded onto the Mouse Cytokine Array C3 (Raybiotech, Norcross, GA) to measure expression levels of cytokines and chemokines. RayBio® Analysis Tool (Raybiotech) was used to correlate the average signal intensities to relative expression levels of cytokines.

### Analysis of infiltrated immune cells

For flow cytometry analysis of infiltrated immune cells, the collected bladder cells were pre-incubated with Fc block (CD16/CD32, Clone 2.4G2, BD), washed, and incubated with appropriate fluorescent-conjugated antibodies. For immunohistochemistry analysis, the tissue sections were incubated with 0.3% H_2_O_2_ for 10 min to block the endogenous peroxidase activity. The tissue sections were then incubated in 5% BSA for 1 h to block non-specific binding sites before incubation with appropriate primary antibodies and HRP-conjugated secondary antibodies. Staining was developed by 3,3′-diaminodbenzidine substrate and the nuclei were counterstained by hematoxylin.

### Statistical analysis

For survival rates, statistical analysis was performed using the log-rank test. For others, statistical significance was assessed by Student’s t-test; p < 0.05 was considered statistically significant.

### Supplemental methods

Details of methods for baculoviral vector construction, cell culturing, *in vitro* & *in vivo* baculoviral transduction, flow cytometry analysis, histological analysis, and immunostaining are provided in the Supplementary Materials and Methods.

## Results

### Baculoviral vectors effectively transduce the mouse bladder and bladder tumors after intravesical instillation

**Figure 1:**
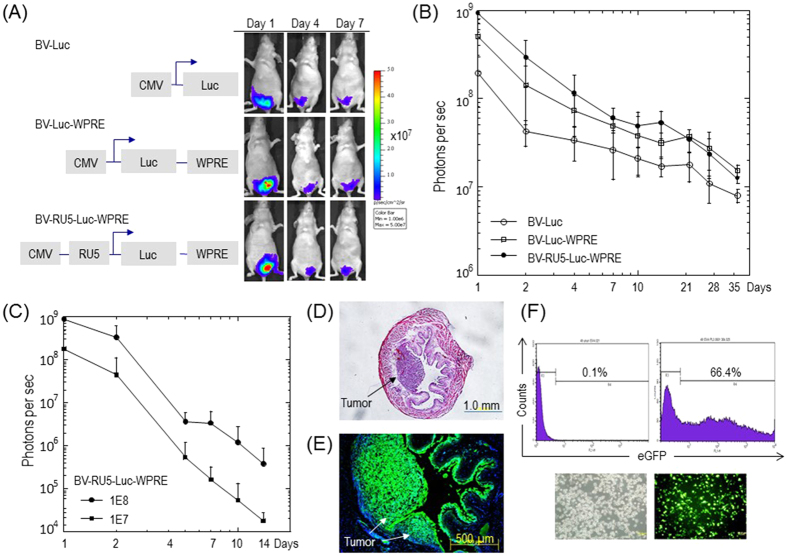
*In vivo* baculoviral transduction after intravesical instillation of baculoviral vectors in mice. (**A**) Bioluminescence images of luciferase reporter gene expression in immunodeficient BALB/c nude mice. Images of representative animals transduced with 3 different baculoviral vectors (10^7^ viral particles per mouse) are shown. Heat map represents the transgene expression area and color represents the intensity. The schematic structures of baculoviral vector expression cassettes are shown on the left. Abbreviations: CMV: the human cytomegalovirus immediate-early gene promoter and enhancer; WPRE: the woodchuck hepatitis virus post-transcriptional regulatory element; RU5: R segment and part of the U5 sequence of the long terminal repeat from the human T-cell leukemia virus type 1. (**B**) Time course analysis of luciferase reporter gene expression in BALB/c nude mice. *In vivo* gene expression levels are quantified by measuring bioluminescence signals. The data represent the mean + s.d., n = 5 per group. (**C**) Time course analysis of luciferase reporter gene expression in C57BL/6 mice. Mice were transduced with the baculoviral vector BV-RU5-Luc-WPRE at a dose of 10^8^ or 10^7^ viral particles per mouse. The data represent the mean + s.d., n = 5 per group. (**D**) MB49 tumor growth in the bladder of C57BL/6 mice. MB49 mouse bladder tumor cells (10^5^ per mouse) were implanted into poly-L-lysine pre-treated bladder and allowed to establish tumors for one week. A bright field image of an H&E stained section showing mouse bladder architecture and a tumor inside the bladder (arrow). (**E**) Immunostaining to demonstrate baculoviral transduction in bladder tumors. Tumor-bearing bladders were collected 24 hours after transduction with BV-RU5-Luc-WPRE for immunostaining with antibodies against the luciferase protein. A fluorescence image is shown. (**F**) Flow cytometric analysis to demonstrate transduction of MB49 tumor cells with BV-RU5-eGFP-WPRE. Phase contrast and fluorescence images of transduced cells are shown in the bottom panels.

We first assessed whether baculovirus could transduce the bladder in immunodeficient BALB/c nude mice. For that, we constructed three different recombinant baculoviral vectors containing a firefly luciferase gene and compared their *in vivo* transduction efficiency after intravesical instillation into the bladder at a dose of 10^7^ viral particles per mouse. The bladders were pre-treated with PLL before viral vector instillation as we found that this pre-treatment significantly enhanced the uptake of baculoviral vectors (Fig. S1A). All three vectors were able to transduce the mouse bladder as evidenced by using the IVIS living animal imaging system (Fig. 1A). One of the baculoviral vectors, BV-RU5-Luc-WPRE, that contains two viral transcriptional regulatory elements WPRE and RU5, provided the highest transgene expression level in the bladder. While decreasing over time, the expression levels provided by the three vectors remained significantly higher than a background level for at least 35 days (Fig. 1B).

We next tested baculoviral transduction in the bladder of immunocompetent C57BL/6 mice after intravesical instillation of BV-RU5-Luc-WPRE. The *in vivo* transduction efficiency was dosage-dependent with the luciferase expression level at day 1 in C57BL/6 mice treated with 10^8^ viral particles per mouse being approximately 5-fold greater than that provided by treatment with 10^7^ viral particles per mouse (Fig. 1C). Although the initial expression level provided by 10^8^ viral particles of BV-RU5-Luc-WPRE in C57BL/6 mice was similar to that observed in immunodeficient nude mice treated with 10^7^ viral particles, the level dropped quickly and the detectable transgene expression lasted for approximately 2 weeks only. The difference in transgene expression between immunodeficient nude mice and immunocompetent mice indicates a strong immune response to baculoviral transduction that might eliminate the viral vectors in the transduced organ. After intravesical instillation with 10^8^ viral particles in C57BL/6 mice we observed no behavioral abnormalities and hepato- and nephro-toxicities (Fig. S2). Hence, this dose was used for all following animal experiments.

We then tested whether BV-RU5-Luc-WPRE, following intravesical instillation, could transduce bladder tumors in an orthotopic tumor model generated by implantation of syngeneic MB49 bladder cancer cells into the bladders of C57BL/6 mice (Fig. 1D). Immunohistological staining with an antibody against the luciferase protein confirmed that the viral vectors transduced the tumors efficiently, penetrating deep into the tumor mass and distributing extensively throughout the whole tumor bed (Fig. 1E). However, in tumor-free areas, as well as in the normal bladder, baculovirus-mediated transgene expression was confined to the superficial bladder epithelium, suggesting a restricted regional transgene delivery by intravesically instilled baculoviral vectors in the normal bladder (Fig. 1E; Fig. S1B). Baculoviral transduction of MB49 mouse bladder cancer cells was further confirmed using BV-RU5-eGFP-WPRE (Fig. 1F). This viral vector could also transduce several of human bladder cancer cell lines, including T24, HBC1, HTB2 and HTB5 (Fig. S3).

### Baculoviral transduction alone is capable of retarding bladder tumor growth

**Figure 2:**
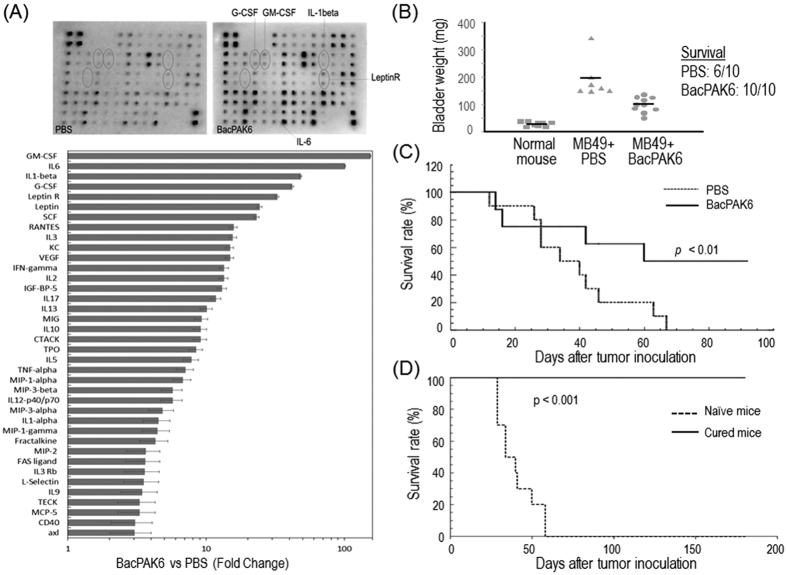
Baculovirus as an immunostimulatory agent for bladder cancer therapy. (**A**) Baculovirus-mediated cytokine/chemokine expression in mice treated with PBS or BacPAK6, a baculoviral vector without mammalian gene expression cassette. *Top:* Bladders were harvested 48 hours after the respective instillation, homogenized and extracts were used to probe the cytokine/chemokine arrays. After densitometric analysis, the top 5 up-regulated cytokines/chemokines are labeled on the blots. The pictures shown are representative. *Bottom:* Densitometric analysis of the cytokine/chemokine arrays. The RayBio® Analysis Tool was used to relate average signal intensity to cytokine/chemokine expression level. The average signal intensity of 3 mice per group and a >3-fold change as a baseline was used for analysis. (**B**) Therapeutic effects of BacPAK6 as demonstrated by bladder weight measurement. Mice were inoculated with MB49 tumor cells intravesically and 7 days later treated with PBS or BacPAK6. Survived mice were sacrificed on day 21 post-tumor inoculation and bladders were collected for weight measurement. The data represent mean + s.d., n = 6 or 10 per group. (**C**) BacPAK6 transduction prolongs survival of bladder tumor-bearing mice. Survival curves till day 95 are shown. n = 10 in the PBS group and n = 20 in the BacPAK6 group. The statistical analysis was performed using the log rank test. (**D**) Induction of protective immunity in mice survived in BacPAK6 group in Fig. 2C. These mice were re-challenged by intravesical tumor inoculation with MB49 cells (n = 10). Age-matched naïve mice were used as controls and inoculated with the same number of MB49 cells (n = 10). Survival curves till day 180 post-tumor inoculation are shown.

As baculoviral transduction in the bladder possibly stimulates local immune responses, we investigated expression of cytokines and chemokines in the organ upon intravesical instillation of baculovirus in C57BL/6 mice. We used BacPAK6, a baculoviral vector without a mammalian gene expression cassette, for this purpose to avoid possible interference by transgene expression. Using an antibody array method, we detected the up-regulation (>3-fold increase in expression) of 61% of the cytokines and chemokines in a murine array (38 out of 62) in the bladder that received intravesical instillation of BacPAK6 two days before as compared to the expression levels in the bladder that received PBS instillation (Fig. 2A). The top 5 up-regulated proteins were granulocyte-macrophage colony-stimulating factor (GM-CSF, 154-fold increase), interleukin-6 (IL-6, 101-fold increase), interleukin-1 beta (IL1-beta, 48-fold increase), granulocyte colony-stimulating factor (G-CSF, 42-fold increase), and leptin receptor (Leptin R, 33-fold increase). A comparison between the normal mouse bladder receiving no treatment and the bladder receiving PBS instillation showed no significant difference in the expression levels of the cytokines and chemokines (data not shown).

The above antibody array results led us to hypothesize that the up-regulation of cytokines and chemokines upon baculoviral transduction would affect bladder tumor growth. To test this, C57BL/6 mice were inoculated with MB49 tumor cells and one week later randomly distributed into two groups (n = 10 per group): one group received single intravesical instillation of BacPAK6 and another group received PBS as an instillation control. Bladders were harvested on day 21 post-tumor inoculation for weight measurement. Indeed, reduced bladder weight as compared with the PBS group, an indication of retarded tumor growth, was observed in the BacPAK6 group (Fig. 2B). We further observed significantly prolonged survival of the bladder tumor-bearing mice after BacPAK6 instillation in the bladder. While all animals in the PBS control group died within 70 days, 50% of the animals in the treatment group were still alive on day 95 post-tumor inoculation (Fig. 2C, p < 0.01 in log-rank test). Furthermore, no therapeutic effects were observed after baculovirus instillation in immunodeficient nude mice (data not shown). These findings indicate that BacPAK6-triggered immune responses are responsible for the observed antitumor effects.

To further test the impact of baculovirus-triggered immune responses on tumor growth, BacPAK6-treated mice that survived from the above animal experiment in Fig. 2C were re-challenged with MB49 tumor cells intravesically. All mice survived for at least 180 days after the 2nd tumor challenge (Fig. 2D). As a control, age-matched naïve mice were challenged with the same number of MB49 cells and all animals died by day 60. Thus, baculovirus instillation in the bladder is able not just to trigger local immune responses, but also promote systemic, adaptive anti-tumor immunity against MB49 tumor cells.

### Baculoviral transduction-mediated immunogene therapy for bladder cancer in mice

To evaluate the potential of using baculovirus as a gene therapy vector, two recombinant baculoviral vectors (BV-CD40L and BV-IL15) armed with either the mouse CD40L or the mouse IL-15 genes were constructed by replacing the luciferase gene in BV-RU5-Luc-WPRE. Baculovirus-mediated CD40L and IL-15 expressions were confirmed by *in vitro* transduction in MB49 cells, followed by Western blot analysis 48 hours post transduction (Fig. S4). Through subcutaneous injection of original MB49 cells, *in vivo* selection/enrichment, and primary tumor cell culturing, we collected faster growing MB49 tumor cells to evaluate the therapeutic effects of BV-CD40L and BV-IL15 (see Supplementary Information). In the orthotopic model generated with aggressive MB49 cells, hematuria, an early sign of tumor growth, was observed in most of the mice as early as one week after tumor cell inoculation. We treated these tumor-bearing mice with one, two or three repeated instillations of viral vectors or control reagents on days 7, 14 and 21 post-tumor implantation respectively. Since BCG treatment is the gold standard for immunotherapy of bladder cancer, it was included for comparison with baculoviral vectors.

**Figure 3:**
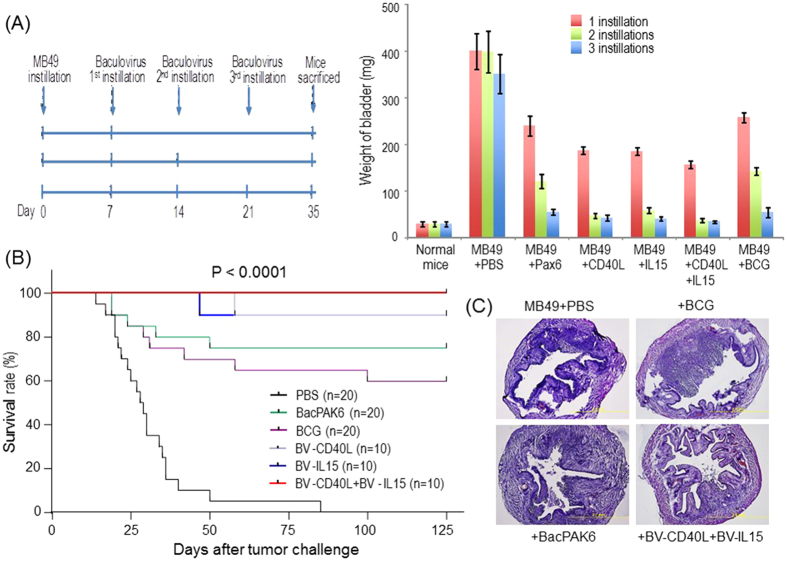
Baculovirus as a gene transfer vector for bladder cancer therapy. (**A**) Bladder weight measurement. *Left*: The animal experiment protocol used. Mice were inoculated with MB49 tumor cells intravesically and subsequently treated three times with baculoviral vectors (days 7, 14, and 21). *Right*: Mice were treated with baculoviral vectors expressing CD40L, IL-15, or both. Control animals were treated with PBS, BCG, or empty baculoviral vector BacPAK6. Mice were sacrificed on day 35 post-tumor inoculation and bladders were collected for weight measurement. The data represent mean + s.d., n = 5 per group. (**B**) Survival curves to demonstrate anti-tumor effects of baculoviral gene therapy after 3 instillations in the mouse bladder. The tumor inoculation and virus/BCG instillations were performed as shown in (**A**). Survival curves till day 125 are shown. The statistical analysis was performed using the log rank test. n = 20 for PBS, BacPAK6, and BCG groups and 10 for BV-CD40L, BV-IL-15 and BV-CD40L+BV-IL-15 groups. (**C**) H&E staining shows MB49 bladder tumor development. Bladders were collected on day 35 post-tumor inoculation.

In the first set of the gene therapy experiments, animals were sacrificed on day 35 post-tumor inoculation and bladders were harvested for weight measurement (Fig. 3A). Therapeutic effects were obvious after one instillation of viral vectors or BCG and became increasingly pronounced with increase in the number of instillations. After giving two or three instillations of both BV-CD40L and BV-IL15, the weight of the bladders from the tumor-inoculated mice was almost same as that in the normal mice group, indicating that multiple instillations of therapeutic gene-expressing baculoviral vectors may promote complete tumor regression. This was supported by a long-term survival study in which all MB49 cell-inoculated mice that were administered three instillations of both BV-CD40L and BV-IL15 survived for at least 12 months. Figure 3B depicts the survival rates of different groups at day 125 post-tumor inoculation, with 100% survival in the group treated with both BV-CD40L and BV-IL15, followed by 90% in the mice receiving BV-CD40L or BV-IL15, 75% in the BacPAK6 group, and 60% in BCG-treated mice. The results of statistical analysis of survival rates are shown in Table S1. Complete tumor regression after three instillations of both BV-CD40L and BV-IL15 was confirmed by histological examination (Fig. 3C). In the mice sacrificed on day 35 post-tumor inoculation bladder tumors were almost undetectable and distinct normal transitional epithelium structure of the bladder mucosa was seen. Taken together, our results confirmed that three instillations of both BV-CD40L and BV-IL15 could effectively suppress bladder cancer growth *in vivo*.

### Baculoviral transduction in the bladder is associated with robust infiltration of immune cells

**Figure 4:**
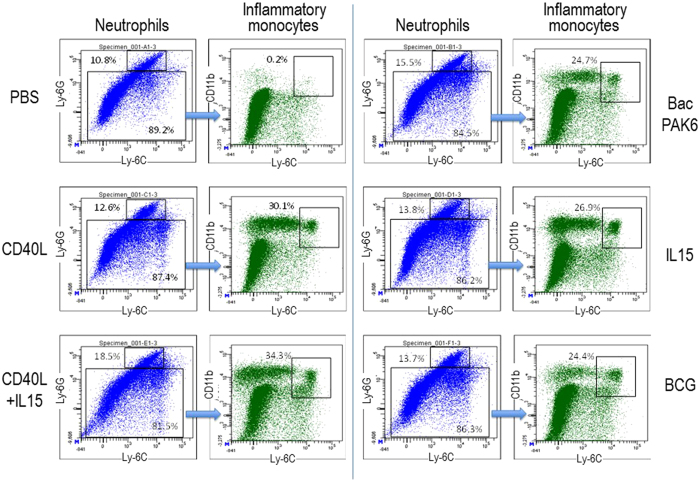
Infiltration of innate immune cells into the bladder after intravesical instillation with PBS, BCG, or baculoviral vectors. Three bladder samples per group were collected 16 hours after the third instillation for flow cytometric analysis. Neutrophils were defined as live CD45.2^+^Ly-6G^+^ cells; inflammatory monocytes were defined as live CD45.2^+^Ly-6G^−^Ly-6C^high^CD11b^+^ cells. Representative FACS plots of two repeated experiments are shown.

To elucidate the cellular mechanisms underlying the immune response activated by baculoviral transduction, we used flow cytometric analysis to characterize immune cell infiltrates in the bladder after repeated instillations. Sixteen-hours after the third instillation as illustrated in Fig. 3A, C57BL/6 mouse bladders were harvested, digested and stained for analysis. Neutrophils were defined as live CD45.2^+^Ly-6G^+^ cells and inflammatory monocytes as live CD45.2^+^Ly-6G^−^Ly-6C^high^CD11b^+^ cells. Results were expressed as % of a lymphocyte subset among live leukocytes (Fig. 4). We observed significantly elevated infiltration of inflammatory monocytes in the bladder after repeated intravesical instillations of BCG, BacPAK6, or baculoviral gene therapy vectors as compared to PBS instillation. The percentage of inflammatory monocytes among live leukocytes increased from 0.2% in the PBS group to approximately 20 to 30% after treatment with BCG, BacPAK6, and baculoviral gene therapy vectors, with the highest infiltration rate (34.3%) after co-instillations of BV-CD40L and BV-IL15. Neutrophil infiltration increased modestly, from 10% in the PBS group to 12 to 18% after treatment with BCG, BacPAK6, or baculoviral gene therapy vectors. Thus, inflammatory monocytes could be one type of effector cells for eliminating bladder tumors by baculoviral transduction in our mouse model.

**Figure 5:**
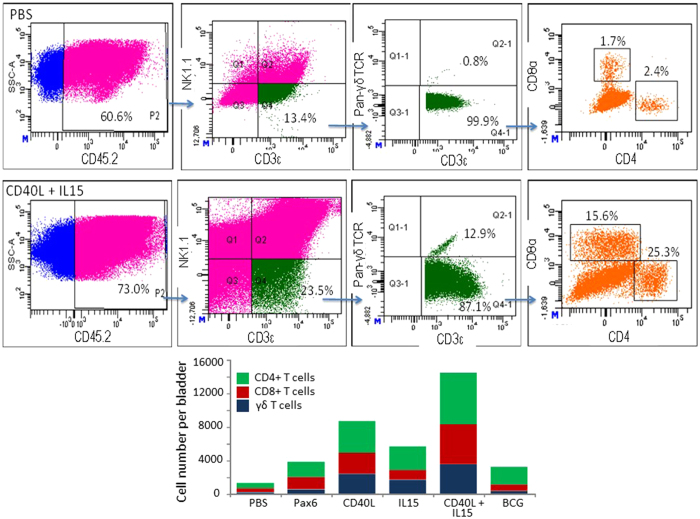
Infiltration of T cells into the bladder after intravesical instillation with PBS, BCG or baculoviral vectors. Three bladder samples per group were collected 16 hours after the third instillation for flow cytometric analysis. T cells were gated as live CD45.2^+^CD3ε^+^NK1.1^−^ cells. The gated T cells were further gated as γδ TCR positive or negative cells, and the latter population was assessed for CD4 or CD8a expression. Representative FACS plots of two repeated experiments are shown. *Bottom:* Average numbers of T cell subpopulations are shown.

**Figure 6:**
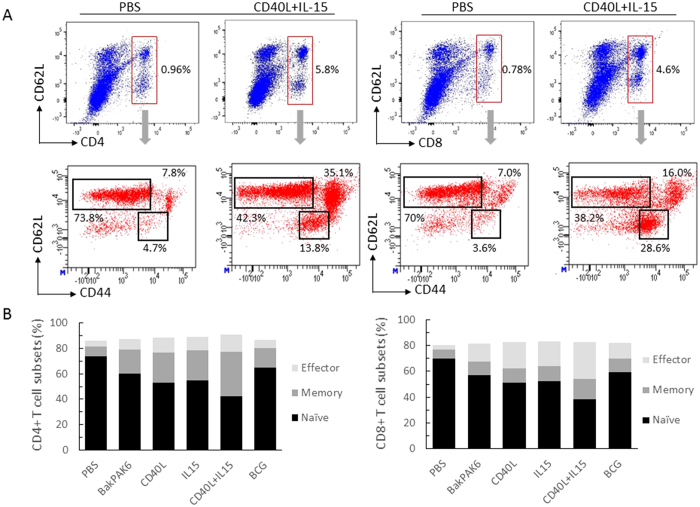
T lymphocyte subset analysis upon repeated intravesical instillations with PBS, baculoviral vectors or BCG. Three mouse bladder samples per group were collected 16 hours after the third instillation and cut into small pieces for enzyme digestion. The collected bladder cells were stained with CD4, CD8, CD62L and CD44 antibodies conjugated with fluorescence, and the results were analyzed by flow cytometry. (**A**) CD4+ (left) and CD8+ (right) cells were gated for T lymphocyte subset analysis. Naïve T cells were defined as CD44lowCD62Lhigh, memory T cells as CD44highCD62Lhigh and effector T cells as CD44highCD62Llow. FACS plots shown are representative of two independent experiments. (**B**) Average numbers of CD4+ (left) and CD8+ (right) T lymphocyte subsets.

T lymphocytes were gated as live CD45.2^+^CD3ε^+^NK1.1^−^ cells, and then as γδ T-cell receptor (TCR) positive or negative cells. From the γδ TCR negative population CD4 and CD8α expression were assessed. As illustrated in Fig. 5, both BCG and BacPAK6 increased the accumulation of T lymphocytes in the bladder, with a slightly higher number of CD8+ T lymphocytes after BacPAK6 instillation. Using BV-CD40L and BV-IL15 for repeated instillations, total T cell populations increased by 2-fold and 1.5-fold respectively as compared with BacPAK6. Co-instillations of BV-CD40L and BV-IL15 increased the accumulation of CD4^+^ T cells and CD8^+^ T cells by approximately 4-fold. Furthermore, co-instillations with BV-CD40L and BV-IL15 generated a profound stimulatory effect on the accumulation of γδ T cells, providing an approximately 10-fold increase in γδ T cell infiltration in the bladder. We further analyzed local T lymphocyte subsets based on the expression of T-cell memory markers CD44 and CD62L (Fig. 6A). Upon repeated intravesical instillations with baculoviral vectors or BCG, the naïve T cells defined as CD44lowCD62Lhigh were markedly decreased, accompanied by increased percentages of CD44highCD62Llow effector T cells and CD44highCD62Lhigh memory T cells in both CD4+ and CD8+ compartments (Fig. 6B). These changes were most obvious after co-instillations with BV-CD40L and BV-IL15. The percentages of effector and memory T cells, which play a pivotal role in the development of immune responses, increased from 12% in the PBS group to 49% in the CD40L+IL15 group in the CD4+ T cell population and 11% in the PBS group to 44% in the CD40L+IL15 group in the in the CD8+ T cell population. Lymphocytic infiltrations after intravesical instillation of BacPAK6 and BV-CD40L/BV-IL15 were also confirmed by immunostaining (Fig. S5). Such pronounced lymphocyte accumulation triggered by baculoviral transduction might play important roles in the above observed bladder tumor regression.

## Discussion

Bladder cancer is the most common form of malignancy in the urinary tract. At initial diagnosis the majority of bladder cancer are NMIBC. The recurrence rate of NMIBC after transurethral resection could be as high as 70%, necessitating adjuvant therapy to control recurrence and progression^1^,^2^. The current study demonstrated for the first time that baculoviral transduction is a possible new approach for adjuvant treatment of bladder cancer. Baculoviruses can be used not only for immune stimulation, but also for therapeutic gene delivery, providing a new combinatorial approach that harnesses the power of immunotherapy and gene therapy in a single viral vector.

Given their intrinsic potent immunostimulatory property and efficient cell transduction capacity, baculoviral vectors offer an unprecedented advantage over many other viral vectors in immunotherapy against bladder cancer. We observed that intravesical instillation of empty baculoviral vectors, without the use of any therapeutic genes, was effective in prolonging the life of mice with established orthotopic bladder cancer. This finding is consistent with previous studies that found that after being injected into the animal body, baculovirus can elicit protective innate immune responses^30^,^35^,^36^,^37^,^38^. This property has been exploited to protect animals from lethal challenges with encephalomyocarditis virus^35^ and influenza virus^37^, suppress liver cirrhosis induced by dimethylnirosamine^38^, and inhibit tumor growth^30^.

Presumably, the initial step after baculovirus instillation in the bladder should be the binding of the virus to the urothelium, after which the virus can enter both normal and malignant cells, resulting in urothelial activation and subsequent inflammatory responses within the bladder. Virus antigens can be presented on the surfaces of transduced urothelial and antigen-presenting cells in the context of MHC class II to stimulate CD4^+^ T cells. Intracellular viral antigens will predominantly induce Th1 immune response, resulting in secretion of Th1-type cytokines. Indeed, we have observed the induction of an array of cytokines by intravesical instillation of baculovirus. These baculovirus-induced cytokines include Th1-type of cytokines such as IFN-gamma, IL-2, TNF-α, and IL12 as well as Th2-type of cytokines IL-13 and IL-10. Although the specific role of each of these cytokines in orchestrating baculovirus-induced anti-tumor immunity is not clear at this point, a high-level expression of Th1 cytokines has been observed to be associated with BCG responders and effective BCG therapy of bladder cancer depends largely on proper induction of the Th1 immune pathway. Baculovirus DNA contains abundant CpG motifs^32^,^33^, and CpG stimulates host production of IL-12 and drives Th1 immune responses, providing potential therapeutic value in treating cancer.

Previous studies have further demonstrated that baculovirus potentiates adaptive immune responses by transducing professional antigen-presenting cells and inducing INF-α and INF-β^28^,^29^,^31^,^39^. The initiation of an adaptive immune response is critically dependent on the activation, functional maturation and migration of dendritic cells. Baculovirus can transduce dendritic cells, activate these cells via the interaction with intracellular toll-like receptor 9, and promote their maturation^28^,^29^,^31^,^32^,^37^,^39^. These immune stimulatory effects can possibly be harnessed to promote systemic anti-tumor immunity. This hypothesis is supported by the current study using a syngeneic model system that provides intact immune functions and allows the study of therapeutic vaccines against bladder cancer. With this mouse model, we demonstrated that tumor-bearing animals that were cured by baculovirus instillation survived after the 2nd tumor challenge.

To test the idea of using wild-type baculovirus as an adjunct in the design of antitumor therapies, Takaku *et al.* have examined the effects of intravenously injected baculoviruses that do not express foreign genes on anti-tumor immunity in immunocompetent mice with B16 mouse melanoma and demonstrated the activation of NK cell-dependent antitumor immunity by baculovirus^30^. Further studies from the same laboratory indicate that baculovirus-induced antitumor action possibly also involves acquired immunity by enhancing tumor-specific cytotoxic T lymphocyte (CTL) responses and tumor-specific antibody production^31^,^39^. These findings highlight that the intrinsic immunostimulatory property of baculovirus can possibly be favorably exploited for cancer immunotherapy. However, since baculoviruses are highly sensitive to the inactivation by serum complement proteins^34^, systemically delivered baculoviral vectors usually do not transduce target cells. In the above studies from Takaku’s lab, high titers of baculoviruses were used to overcome the ability of the complement to neutralize the viruses, yet there was no demonstration of baculovirus-mediated transgene expression^30^,^31^,^39^.

As shown in the current study, the efficacy of baculovirus-mediated bladder cancer therapy was further improved by including therapeutic genes into baculoviral vectors. In this regard, we showed the strong anti-tumor effects of baculoviral vector-mediated CD40L or IL-15 expression in an animal model with aggressively growing bladder cancer. Local transduction, either in normal or malignant bladder cells, that sufficiently activates only targeted cytokines would be an attractive strategy to improve the efficiency of immunotherapy and to diminish possible side effects associated with systemic exposure. Using an intravesical liposomal gene delivery approach in a mouse bladder cancer model, IL-15 gene therapy has been demonstrated to be a new promising approach for bladder cancer treatment^40^. Adenoviral vectors encoding IL-15 were also found to be able to abolish tumorigenicity of murine bladder tumor MB49^41^. Following preclinical testing in experimental bladder cancer animal models, a Phase I/IIa clinical trial using adenoviral vector expressing CD40L for immunogene therapy of bladder cancer has been performed and demonstrated a boosted immune activation^14^.

CD40L, also called CD154, is a potent Th1 immune stimulator. CD40L is mainly expressed on activated CD4^+^ T cells and interacts with CD40 expressed on a wide range of antigen-presenting cells (APCs) and malignant cells^42^. CD40/CD40L ligation may activate APCs, stimulating their maturation to present antigens to T cells and ensuring the generation of antigen-specific CTLs. CD40L stimulation can also activate secretion of cytokines, such as IL-12 and IFN-γ, leading to a Th1 response, and abrogate the suppressive effect of T regulatory cells. Moreover, CD40 expression has been found on various tumor cells, and CD40/CD40L ligation can inhibit proliferation and induce apoptosis directly in tumor cells by activation of NF-κB, AP-1, CD95, or caspase-depended pathways^43^. Acting both as an activator of immune cells and cancer cell death inducer, CD40L is currently under intensive investigation for its potent anti-tumor effects.

IL-15 is a 15-kDa cytokine member in the IL-2 family. While IL-15 has pleiotropic immune-enhancing activities, it plays a pivotal role in the generation and maintenance of memory CD8^+^ T cells and NK cells^44^. A first-in-human Phase I clinical trial that involved recombinant IL-15 in patients with refractory metastatic malignant melanoma and metastatic renal cell cancer has just completed^45^. The study revealed that NK cells and γδ T lymphocytes in blood were most dramatically affected, followed by CD8^+^ memory cells, by IL-15 administration. Since IL-15 activates anti-tumor effectors such as NK cells, γδ T lymphocytes, and memory phenotype CD8^+^ T cells and exerts more long-lasting antitumor effects, it might be especially suitable for immunotherapy of NMIBC.

When viewed from the interaction perspective, IL-15 can induce CD40 expression on conventional DCs and interaction between CD40 on conventional DCs and CD40L on plasmacytoid DCs leads to IL-12 production by conventional DCs, which is essential for CpG-induced immune activation^46^. On the other hand, stimulation of monocytes or DCs with the ligation of CD40 with CD40L or an agonistic anti-CD40 monoclonal antibody can coordinately induce IL-15 and IL-15Ra expression. IL-15Ra on the cell surfaces of monocytes or DCs can then present IL-15 *in trans* to cells such as CD8^+^ memory T cells and NK cells that express IL-2/IL-15Rb^47^. Also, IL-15 can cooperate with CD40L to increase growth of normal and follicular lymphoma B-cells^48^. Although the mechanism of their interaction is still unclear, the baculoviral transduction-mediated co-expression of IL-15 and CD40L, as demonstrated in the current study, exhibits potent therapeutic efficacy for bladder cancer.

The bladder is a confined compartment in which an immunotherapy agent can be given in a high concentration to effectively recruit immune cells and activate them locally, thus serving as an ideal target organ for immunotherapy. Normally, the bladder can be regarded as an organ that is not infiltrated by large numbers of immune cells^3^,^49^. This study shows that following intravesical baculoviral transduction, the bladder is infiltrated by mononuclear leukocytes, including inflammatory monocytes, CD4^+^, CD8^+^, and γδ T lymphocytes. We focused mainly on immune cell infiltration after repeated instillations since this is a clinically relevant scheme. This infiltration pattern of immune cells after repeated instillations of baculoviral vectors is in general similar to the one observed following intravesical BCG instillation^3^,^49^. However, instillation with baculoviral vectors expressing CD40L and IL-15 significantly increased the influx of CD3^+^ T lymphocytes with higher absolute numbers of CD4^+^, CD8^+^, and γδ T lymphocytes. This increase is much more pronounced when looking at γδ T lymphocytes, the non-MHC-restricted cytotoxic cells that can initiate cytotoxicity after infection, even though BCG is known to be able to induce the proliferation of γδ T lymphocytes^50^. Also noteworthy, intravesical instillation of baculovirus alone was able to increase the number of γδ T lymphocytes in the bladder. While the antitumor activity of baculoviral transduction in the current bladder cancer model possibly depends on the interplay of different immune cells, the correlation between increased numbers of γδ T lymphocytes in the bladder and anti-cancer efficacies achieved with different therapeutics used for bladder instillation indicates that this type of cytotoxic effector cells play an important role in treatment success.

In conclusion, we have demonstrated in a syngeneic orthotopic animal model of bladder cancer that insect baculovirus can be used as a new agent for bladder cancer therapy with such functions as stimulating local innate immune reactions, promoting systemic anti-tumor immune responses, and delivering therapeutic genes. When tested as an adjuvant therapy for urothelial malignancies, the success of the approach will benefit bladder cancer patients who fail conventional BCG immunotherapy or who are intolerant of BCG treatment. When used as a single therapy, this intravesical immunotherapy approach may improve bladder preservation and the quality of life for patients, thus delivering better health outcomes. Hence, intravesical instillation of recombinant baculoviral vectors holds potential to develop into an advanced therapy for NMIBC.

## Additional Information

**How to cite this article**: Ang, W. X. *et al.* Local Immune Stimulation by Intravesical Instillation of Baculovirus to Enable Bladder Cancer Therapy. *Sci. Rep.* **6**, 27455; doi: 10.1038/srep27455 (2016).

## Supplementary Material

Supplementary Information
