# Supplementary material for: Local Immune Stimulation by Intravesical Instillation of Baculovirus to Enable Bladder Cancer Therapy
Source: Sci Rep. 2016 Jun 8;6:27455. doi: 10.1038/srep27455 (PMC4897700; doi:10.1038/srep27455)
Supplement: Supplementary Information [file srep27455-s1.pdf]

Local Immune Stimulation by Intravesical Instillation of Baculovirus to Enable Bladder Cancer Therapy

Wei Xia Ang[1,2], Ying Zhao[2], Timothy Kwang[3], Chunxiao Wu[2,3], Can Chen[3], Han Chong Toh[4], Ratha Mahendran[5], Kesavan Esuvaranathan[5], and Shu Wang[1, 2*]

[1]Department of Biological Sciences, National University of Singapore, Singapore 117543
[2]Institute of Bioengineering and Nanotechnology, Singapore 138669
[3]Tessa Therapeutics, Singapore 239351
[4]Department of Medical Oncology, National Cancer Centre, Singapore 169610
[5]Department of Surgery, Yong Loo Lin School of Medicine, National University of Singapore, Singapore 119074

SUPPLEMENTARY INFORMATION

Supplementary Materials and Methods

Baculoviral vectors

Recombinant baculoviral vectors, CMV-Luc, CMV-Luc-WPRE and CMV-RU5-Luc-WPRE, were constructed using BAC-to-BAC baculovirus expression system (Invitrogen, Carlsbad, CA). CMV-Luc contains a luciferase gene under the control of human cytomegalovirus (CMV) early promoter. CMV-Luc-WPRE has an extra woodchuck hepatitis virus posttranscriptional regulatory element (WPRE) at the 3' untranslated region (UTR). CMV-RU5-Luc-WPRE has another regulatory element RU5, the R segment and part of the U5 sequence of long terminal repeat from the human T-cell leukemia virus type 1, at the 5' UTR. These luciferase-expressing viruses were produced by transfection of Sf9 insect cells using corresponding bacmids. BV-CD40 ligand (CD40L) virus and BV-IL15 virus which contain the mouse CD40L gene and mouse IL15 gene respectively under the control of CMV promoter with RU5 at 5' UTR and WPRE at 3' UTR, were produced by homologous recombination after co-transfection of *Sf9* insect cells with pBacPAK6 transfer vector containing the expression cassette and linearized AcMNPV viral DNA (Clontech, Mountain View, CA). BacPAK6, the parental virus with the lacZ gene driven by viral polyhedrin promoter, was obtained from Clontech. Recombinant baculoviruses were amplified in *Sf9* cells at an MOI of 0.1 and the virus-containing supernatant was collected 3 days after virus infection. Viruses were pelleted down at 28,000 g for 1 hour and re-suspended in PBS.

Cell lines and in vitro baculoviral transduction

Sf9 insect cells (Invitrogen) were maintained in sf-900 III serum free medium (Invitrogen). Murine bladder carcinoma cell line MB49 was a gift from Dr. Esuvaranathan (National University Hospital, Singapore). The human bladder carcinoma cell lines T24, HBC1, HTB2 and HTB5 were purchased from ATCC. The cell lines were maintained in RPMI 1640 supplemented with 10% fetal bovine serum (Hyclone, Logan, UT), 2 mM L-Glutamine, 1% penicillin and streptomycin (Sigma-Aldrich, St. Louis, MO) at 37°C and 5% CO2. For *in vitro* baculoviral transduction experiments, the tumor cells were

incubated with baculoviral vectors at an MOI of 100 overnight at 37°C. Transgene expression was determined 24 hours after transduction.

In vivo baculoviral transduction

Adult female BALB/c nude mice and adult female C57BL/6 mice (weight 20 g, aged 5-6 weeks) were used to evaluate *in vivo* transduction efficiency in the bladder. Mice were anaesthetized (150 mg/kg ketamine and 10 mg/kg xylazine) and catheterized. A 24-guage catheter (BD Medical, Franklin Lakes, NJ) was carefully inserted into the bladder of anesthetized female mice through urethra. After the residual urine was squeezed out, the bladder was infused with 100 μl of 10 μg/ml PLL. The PLL solution was retained in the bladder for 30 min before being squeezed out. The bladder was then washed with 100 μl of PBS. After the PLL pre-treatment, baculoviral vectors in PBS (1 x 10^7 pfu viral particles or 1 x 10^8 pfu viral particles in 100 μl) was instilled and retained in the bladder for 2 hours before the catheter was removed. To evaluate *in vivo* transduction efficiency in the bladder, mice transduced with a baculoviral vector with the luciferase reporter gene were anaesthetized, i.p. injected with 150 mg/kg luciferin (Promega, Madison, WI), and imaged in the supine position with the IVIS100 imaging system coupled with a cool CCD camera and an emission filter of 560 nm (Caliper Life Sciences, Hopkinton, MA). Images and the luminescent signals were acquired and analyzed with the Xenogen living imaging software v2.5 and quantified as photons per second. All handling and care of animals were carried out according to the Guidelines on the Care and Use of Animals for Scientific Purposes issued by the National Advisory Committee for Laboratory Animal Research, Singapore.

Generate MB49 bladder cancer cells with increased malignant potential through in vivo selection

To evaluate baculoviral transduction-mediated immunogene therapy, adult female C57BL/6 mice were used to generate orthotopic bladder tumors on the luminal surface of the bladder by intravesical instillation of syngeneic MB49 cells. Faster growing sublines of MB49 cells were first established by in vivo selection[1,2]. C57BL/6 mice were given a single subcutaneous injection of 5 x 10^5 MB49 cells in the right hind flank region. Mice were then closely observed for tumor growth. The progressively enlarged tumor tissues were aseptically removed once reached the size of 2 cm. Tumor tissues were immediately minced into small pieces in cold PBS and digested in Collagenase D (1 mg/ml; Roche) in RPMI for 30 min at 37 °C. Digested tissues were further pressed through a 40 µm cell strainer before being subject to culture in vitro to establish re-cultivated MB49 cells. These MB49 cells re-cultured from the established tumors showed an increased malignant potential when reinjected into animals and hematuria was observed in most of the mice as early as one week after bladder inoculation of the tumor cells.

Flow cytometry analysis of infiltrated immune cells

For flow cytometry analysis of infiltrated immune cells, mouse bladders were dissected, cut into small pieces, and then digested in DMEM (Invitrogen) containing collagenase D (1 mg/ml) (Roche), liberase TM (0.17 U/ml) (Roche), and deoxyribonuclease 1 (1 U/ml) (Invitrogen) at 37°C for 1 hour. The digested

tissue suspensions were filtered through a 70-μm cell strainer to remove debris. After further filtration through a 40-μm cell strainer, the collected bladder cells were pre-incubated with Fc block (CD16/CD32, Clone 2.4G2, BD), washed, and incubated with appropriate fluorescent-conjugated antibodies: CD45.2 (clone 104), CD3e (clone 145-2C11), NK1.1 (clone PK136), CD8a (clone 53-6.7), CD44 (clone IM7), CD45RA (clone 14.8), Ly-6C (clone AL-21), and Ly-6G (clone AL-21) antibodies were from BD. CD4 (clone GK1.5), CD11b (clone M1/70), pan-γδ TCR (clone GL3), and CD62L (clone MEL-14) were from eBioscience (San Diego, CA).

Histological analysis and immunostaining

For histological examination, bladders were harvested and fixed in 4% paraformaldehyde overnight, suspended in 30% sucrose, and embedded in a tissue freezing medium. Cryostat sections at 10 μm were prepared and stained with hematoxylin & eosin (H & E). For immunostaining, the tissue sections were washed twice with Tris-buffered saline Tween-20 (TBST) and incubated in 0.025% Triton X-100 for 10 min. The tissue sections were then incubated in 5% bovine serum albumin (BSA) for 1 h to block nonspecific binding. The rabbit polyclonal anti-luciferase antibody (1:100, Abcam, Cambridge, UK) was applied overnight at 4°C. After 3 times washing with TBST, slides were incubated with the secondary antibody, FITC conjugated goat anti-rabbit IgG (1:200), for 1 hour at room temperature. For immunohistochemistry analysis, the tissue sections were washed twice with TBST and incubated in 0.025% Triton X-100 for 10 min. The tissue sections were incubated with 0.3% H2O2 for 10 min to block the endogenous peroxidase activity. The tissue sections were then incubated in 5% BSA for 1 h to block non-specific binding sites. The rat monoclonal anti-CD8 antibody (1:100, Abcam), rat monoclonal anti-F4/80 antibody (1:100, Abcam), rat monoclonal anti-CD4 antibody (1:100, Abcam), or rabbit polyclonal anti-CD3 antibody (1:100, Abcam) was applied overnight at 4°C. After three times washing with TBST, slides were incubated with the secondary antibody, HRP-conjugated goat anti-rat IgG (1:200) or HRP-conjugated goat anti-rabbit IgG (1:200) for 1 h at room temperature. Staining was developed by 3,3'-diaminodbenzidine substrate and the nucleus was counterstained by hematoxylin.

References

1. Barut, B., A., Klaunig, J., E., Isolation and characterization of metastatic sublines from a murine transitional cell bladder carcinoma. *Clin Exp Metastasis.* **4.** 1-11 (1986).

2. Mueller, M.,M., *et al.* Tumor progression of skin carcinoma cells in vivo promoted by clonal selection, mutagenesis, and autocrine growth regulation by granulocyte colony-stimulating factor and granulocyte-macrophage colony-stimulating factor. *Am J Pathol.* **159.**1567-79 (2001).

Supplementary Table 1. Log-rank test of Kaplan–Meier survival curves

	Statistic	p-value	Significant
PBS v.s. BCG	20.35	6.40E-06	YES
PBS v.s. BacPAK6	24.12	9.00E-07	YES
PBS v.s. BV-CD40L	24.62	7.00E-07	YES
PBS v.s. BV-IL15	23.84	1.00E-06	YES
PBS v.s. BV-CD40L + BV-IL15	25.61	4.20E-07	YES
BCG *v.s.* BacPAK6	0.86	0.3532	NO
BCG *v.s.* BV-CD40L	2.82	0.0928	NO
BCG *v.s.* BV-IL15	2.74	0.0976	NO
BCG *v.s.* BV-CD40L + BV-IL15	4.94	0.0262	YES
BacPAK6 *v.s.* BV-CD40L	1.02	0.3136	NO
BacPAK6 *v.s.* BV-IL15	0.95	0.3286	NO
BacPAK6 *v.s.* BV-CD40L + BV-IL15	2.8	0.0941	NO
BV-CD40L *v.s.* BV-IL15	0	0.9703	NO
BV-CD40L *v.s.* BV-CD40L + BV-IL15	1	0.3173	NO
BV-IL15 *v.s.* BV-CD40L + BV-IL15	1	0.3173	NO
All 6 groups	72.79	<0.0001	YES

Supplementary Figures

Figure S1. Baculoviral transduction in the normal mouse bladder. (A) Effects of poly-L-lysine (PLL) on *in vivo* baculoviral transduction efficiency after intravesical instillation of BV-Luc in BALB/c nude mice and C57BL/6 mice. Pre-treatment of the bladders significantly increased the transduction efficiency. (B) Immunostaining to demonstrate baculoviral transduction in the normal mouse bladder. Transduction was performed by intravesical instillation of BV-RU5-Luc-WPRE in PLL pre-treated bladders. Bladders were collected 24 hours later for immunostaining with antibodies against the luciferase protein. A low-magnification image (left) and a high-magnification image (right) are shown. Note that baculovirus-mediated transgene expression was confined to the superficial bladder epithelium, suggesting a restricted regional transgene delivery by intravesically instilled baculoviral vectors in the normal bladder.

Figure S2. Evaluation of possible hepato- and nephro-toxicities after intravesical instillation of baculoviruses. To determine possible off-target toxic effects associated with intravesical instillation of baculoviruses, 100 μL blood sample was collected from each mouse using orbital sinus blood sampling 24 hours later or one week after intravesical instillation of 10^8 BacPAK6 viral particles or PBS in C57BL/6 mice. The blood concentrations of aspartate aminotransferase (AST), used as a liver damage indicator, and creatinine concentration, used as a kidney damage indicator, were measured. n = 6 mice per group. There are no statistically significant differences between the PBS group and the BacPAK6 group as determined by analysis of variance.

Figure S3. Baculoviral transduction of bladder cancer cells. (A) Effects of poly-L-lysine (PLL) on in vivo baculoviral transduction of MB49 mouse bladder tumors after intravesical instillation of BV-Luc in C57BL/6 mice. MB49 cells were pre-labeled with a lipophilic, near-infrared fluorescent dye DiR. The results showed that baculovirus can transduce bladder cancer cells without the pre-treatment with PLL. (B) Transduction efficiency of BV-GFP in human bladder cancer cells. Left: Representative flow cytometry histograms of GFP expression 24 hours after baculovirus transduction in human bladder cancer cell lines, T24, HBC1, HTB2, and HTB5. Right: Quantitative comparison. Data are average and s.d (3 independent studies conducted for each cell line).

Figure S4. Baculovirus-mediated CD40L and IL-15 protein expressions in MB49 mouse bladder cancer cells. (A) The schematic structures of baculoviral vector expression cassettes. Abbreviations: CMV: the human cytomegalovirus immediate-early gene promoter and enhancer; WPRE: the woodchuck hepatitis virus post-transcriptional regulatory element; RU5: R segment and part of the U5 sequence of long terminal repeat from the human T-cell leukemia virus type 1. (B) Western blot analysis to demonstrate the CD40L and IL-15 protein expressions. MB49 cells were pre-treated with or without poly-L-lysine (PLL) before transduction with either BV-CD40L or BV-IL15 at an MOI of 100 overnight at 37°C. Whole-cell lysates were prepared 48 hours after transduction. The results confirmed again that baculovirus can transduce bladder cancer cells without the pre-treatment with PLL.

Figure S5. Immunostaining to demonstrate lymphocytic infiltration after intravesical instillation of baculoviral vectors BakPAK6 and BV-CD40L/IL15. Normal C57BL/6 mice were treated intravesically with either PBS or the viruses weekly for 3 weeks. Mice were sacrificed 16 hours after the third instillation and bladder sections were stained for immune infiltrates. We observed significantly increased $CD3^{+}$, $CD4^{+}$ and $CD8^{+}$ T lymphocytes and $F4/F80^{+}$ macrophages after BacPAK6 and BV-CD40L/IL15 instillation, suggesting that these cells are likely effector cells mediating the observed tumor regression.

Figure S1

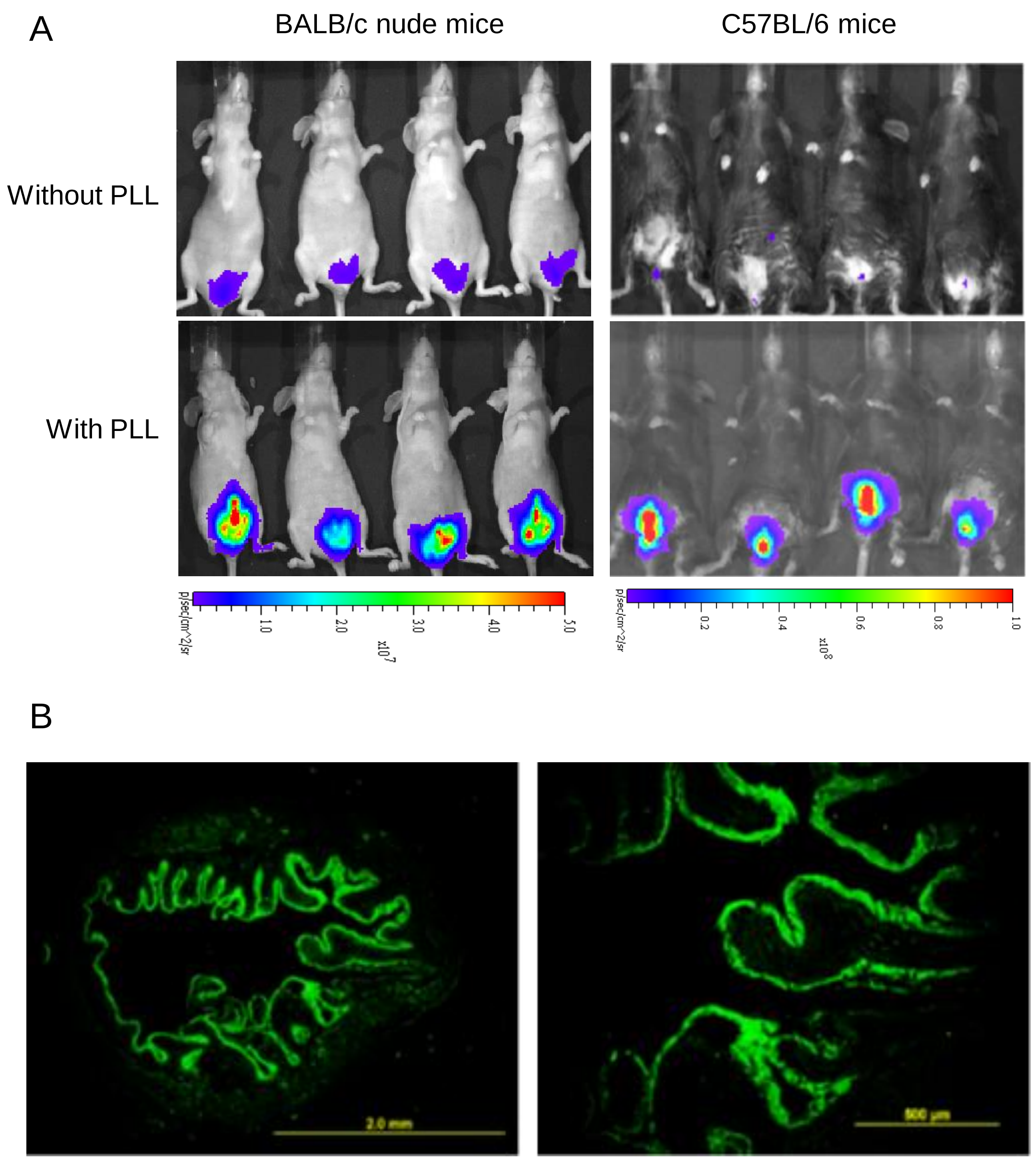

Figure S2

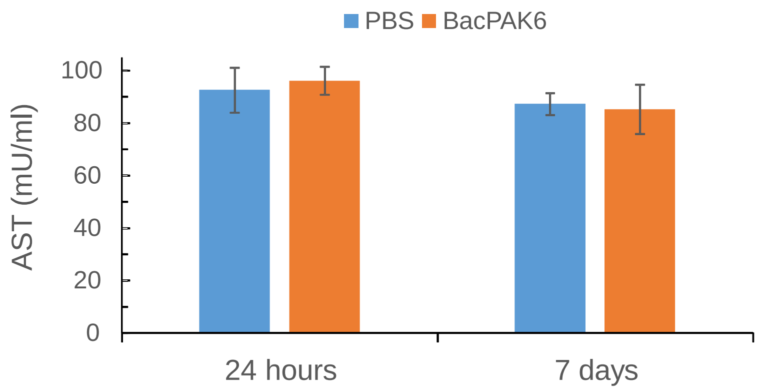
PBS
BacPAK6
AST (mU/ml)
100
80
60
40
20
0
24 hours
7 days

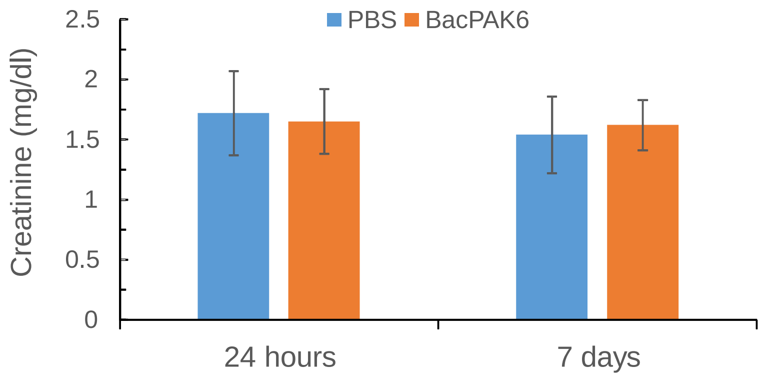
PBS
BacPAK6
Creatinine (mg/dl)
2.5
2
1.5
1
0.5
0
24 hours
7 days

Figure S3

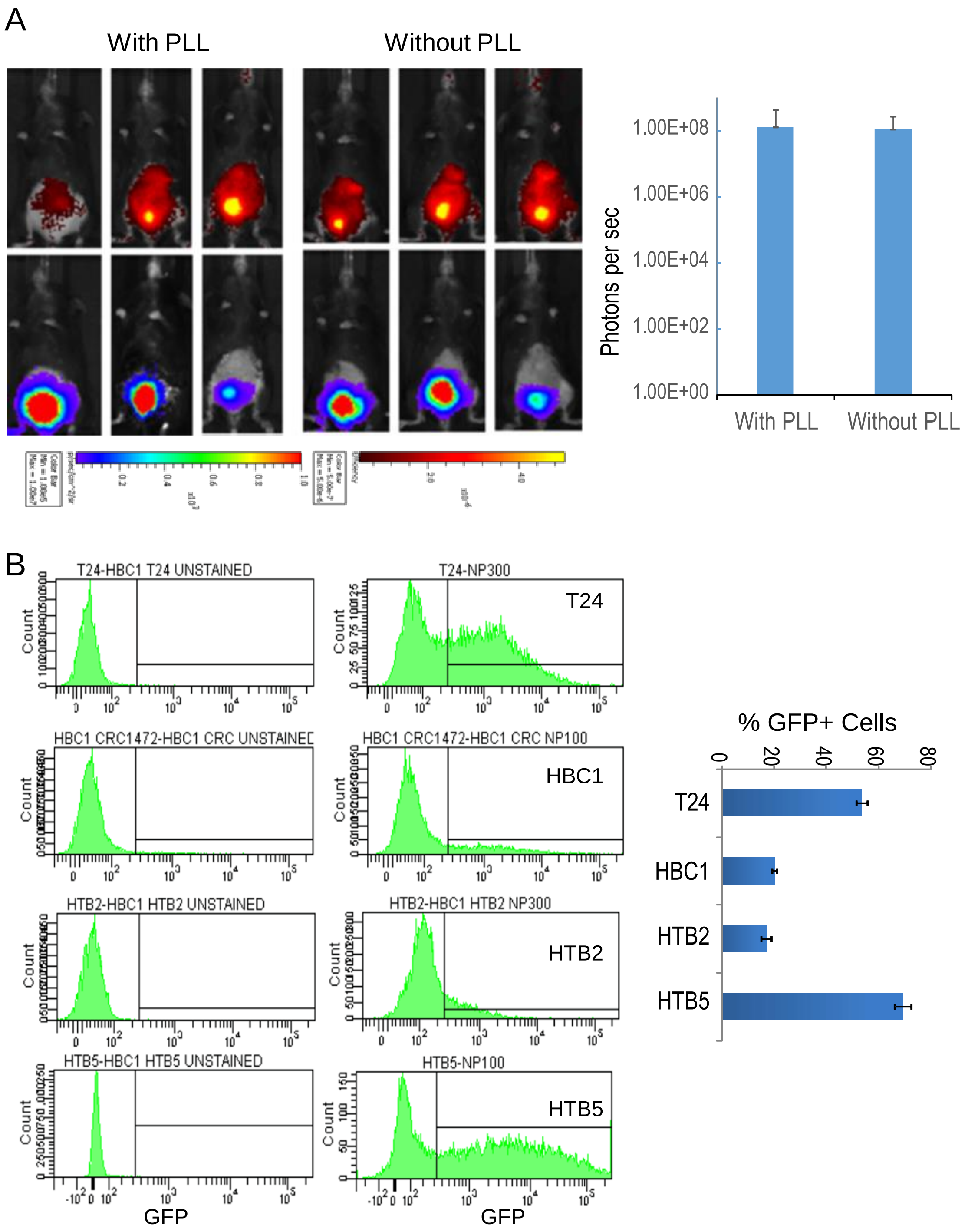
A
With PLL
Without PLL
Photons per sec
1.00E+08
1.00E+06
1.00E+04
1.00E+02
1.00E+00
With PLL
Without PLL
B
T24-HBC1 T24 UNSTAINED
T24-NP300
T24
HBC1 CRC1472-HBC1 CRC UNSTAINED
HBC1 CRC1472-HBC1 CRC NP100
HBC1
HTB2-HBC1 HTB2 UNSTAINED
HTB2-HBC1 HTB2 NP300
HTB2
HTB5-HBC1 HTB5 UNSTAINED
HTB5-NP100
HTB5
Count
GFP
% GFP+ Cells
0
20
40
60
80
T24
HBC1
HTB2
HTB5

Figure S4

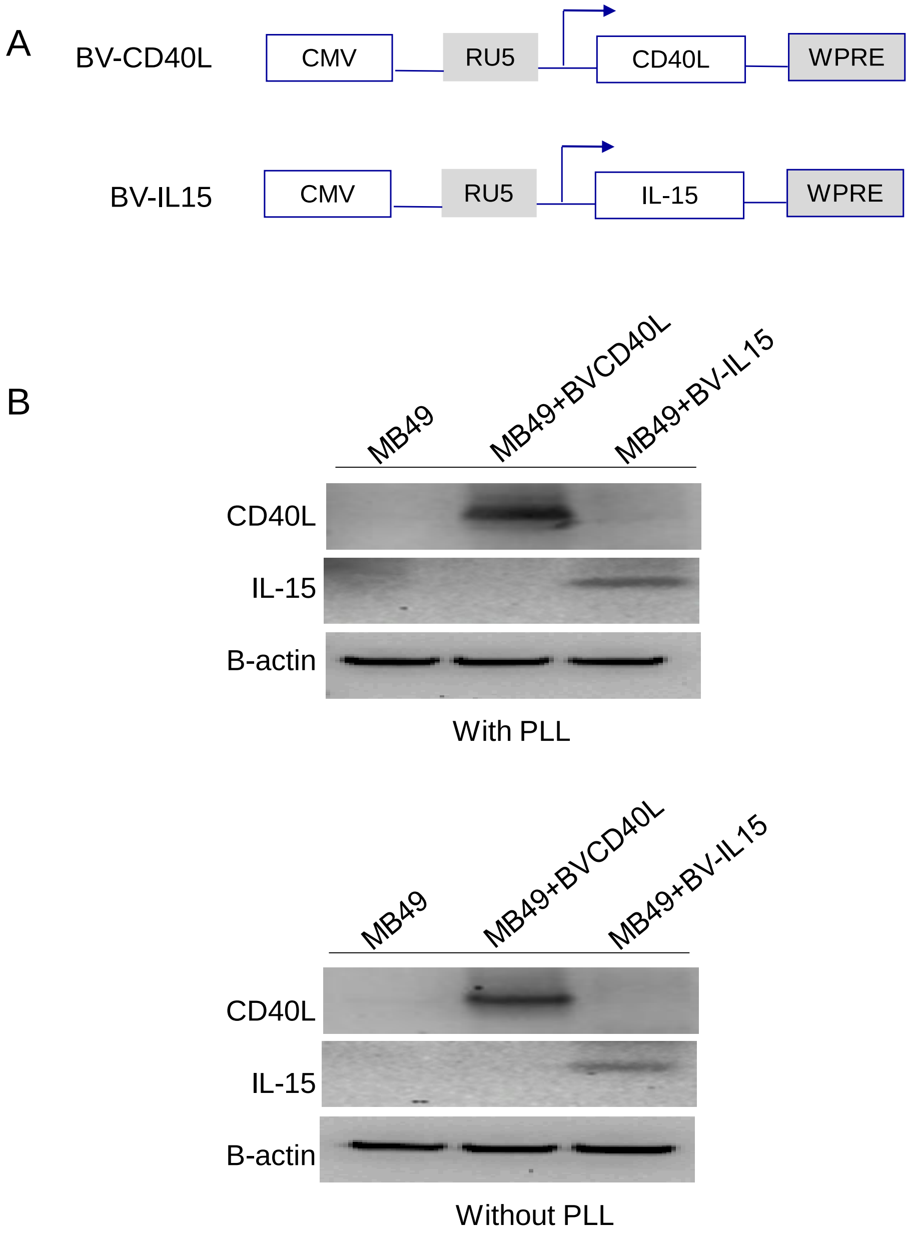
A
BV-CD40L
CMV
RU5
CD40L
WPRE
BV-IL15
CMV
RU5
IL-15
WPRE
B
MB49
MB49+BVCD40L
MB49+BV-IL15
CD40L
IL-15
B-actin
With PLL
MB49
MB49+BVCD40L
MB49+BV-IL15
CD40L
IL-15
B-actin
Without PLL

Figure S5

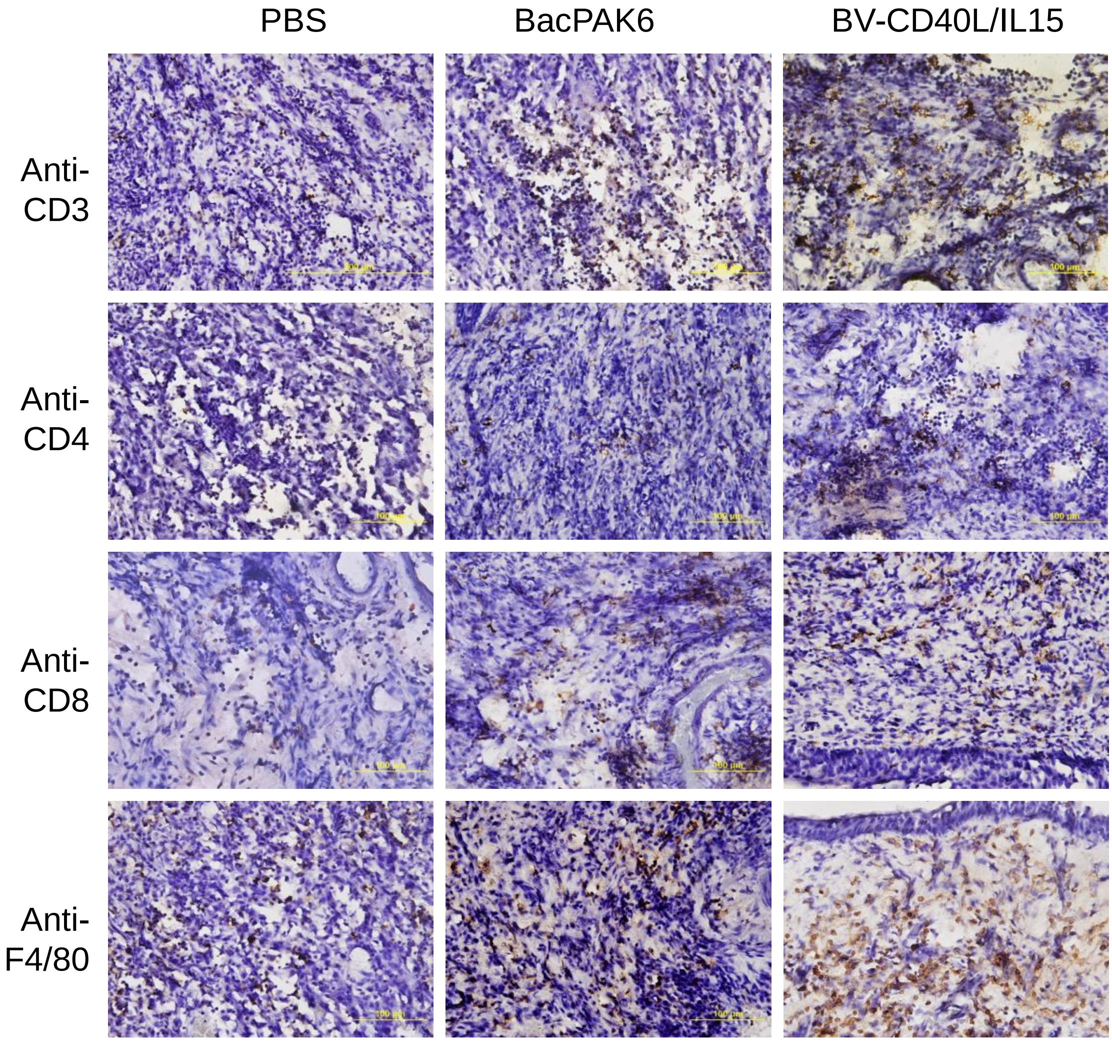
PBS
BacPAK6
BV-CD40L/IL15
Anti-CD3
Anti-CD4
Anti-CD8
Anti-F4/80
